# Risk Factors Associated with Postoperative Outcomes in Diverticular Disease Undergoing Elective Colectomy—A Retrospective Cohort Study from the ACS-NSQIP Database

**DOI:** 10.3390/jcm12237338

**Published:** 2023-11-27

**Authors:** Wan-Hsiang Hu, Samuel Eisenstein, Lisa Parry, Sonia Ramamoorthy

**Affiliations:** 1 Department of Colorectal Surgery, Kaohsiung Chang Gung Memorial Hospital and Chang Gung University College of Medicine, Kaohsiung 833, Taiwan; gary.hu0805@gmail.com; 2 Graduate Institute of Clinical Medical Science, College of Medicine, Chang Gung University, Kaohsiung 333, Taiwan; 3 Department of Surgery, University of California, San Diego Health System, La Jolla, CA 92103, USA; seisenstein@mail.ucsd.edu (S.E.); lparry@mail.ucsd.edu (L.P.); 4 Rebecca and John Moores Cancer Center, University of California, San Diego Health System, La Jolla, CA 92103, USA

**Keywords:** diverticular disease, elective colectomy, postoperative complication, American College of Surgeons National Surgical Quality Improvement Program

## Abstract

Recommendations for elective colectomies after recovery from uncomplicated acute diverticulitis should be individualized. The kinds of associated risk factors that should be considered for this approach remain undetermined. The aim of this study was to identify the risk factors associated with postoperative outcomes in patients with diverticular disease after receiving an elective colectomy. This is a retrospective study using the multi-institutional, nationally validated database of the American College of Surgeons-National Surgical Quality Improvement Program (ACS-NSQIP). The patients who were diagnosed with diverticular disease and received an elective colectomy were included in our risk factor analyses. Postoperative mortality, morbidity, and overall complications were measured. Univariate and multivariate analyses were used to demonstrate the risk factors. We analyzed 30,468 patients with diverticular disease, 67% of whom received an elective colectomy. The rate of 30-day mortality was 0.2%, and superficial surgical site infection was the most common postoperative morbidity (7.2%) in the elective colectomies. The independent risk factors associated with overall complications were age ≥ 75, BMI ≥ 30, smoking status, dyspnea, hypertension, current kidney dialysis, chronic steroid use, ASA III, and open colectomy. In laparoscopic colectomy, 67.5% of the elective colectomies, the associated risk factors associated with overall complications still included age ≥ 75, smoking, hypertension, chronic steroid use, and ASA III. Identification of patient-specific risk factors may inform the decision-making process for elective colectomy and reduce the postoperative complications after mitigation of those risk factors.

## 1. Introduction

In the United States’ population, diverticular disease accounts for close to 300,000 hospital admissions and 1.5 million days of inpatient care annually [1]. The rate of admission for acute diverticulitis increased by 26% from 1998 to 2005 [2]. The estimated cost of treatment is over 2.6 billion per year [3]. Although most of the patients with acute diverticulitis respond to nonoperative treatment, 10–30% require urgent colectomy [4,5], which is accompanied by a high rate of subsequent colostomy or postoperative mortality [6,7].

Prophylactic colectomy was initially recommended for patients after attacks of acute diverticulitis to prevent recurrence and decrease the rate of colostomies [8,9]. Studies of trends in diverticulitis management in the United States show that use of elective colectomy has increased [2,10], and this trend was associated with a wider use of the laparoscopic approach. More recent studies, however, have caused the medical community to reconsider this policy. After elective colectomy, 25% of patients with diverticulitis still had ongoing symptoms [11]. For the majority of patients with complicated diverticulitis who sought an elective colectomy after their first episode, mortality rates were reduced [12]. An elective colectomy after the fourth episode was associated with lower healthcare costs and mortality rates [6]. The recurrence rate of diverticulitis following an elective colectomy was 2.6% to 10% [13,14]. The recommendation of an elective colectomy after uncomplicated diverticulitis has been modified so that this decision is made on an individualized, case-by-case basis [15,16]. Risk factors associated with development of diverticular disease, including obesity, metabolic syndrome, and non-alcoholic liver disease, have been reported [17,18,19]. However, the literature discussing the preoperative risk factors associated with the outcomes of elective colectomy for diverticular disease is scarce [20].

The American College of Surgeons-National Surgical Quality Improvement Program (ACS-NSQIP) database collects preoperative comorbidity and postoperative mortality and morbidity data from patients who received surgery in the hospitals of the United States and Canada. The purpose of this study was to use this large database to demonstrate the risk factors which were associated with the postoperative outcomes of elective colectomies for patients with diverticular disease.

## 2. Materials and Methods

### 2.1. Study Design and Population

**Figure 1:**
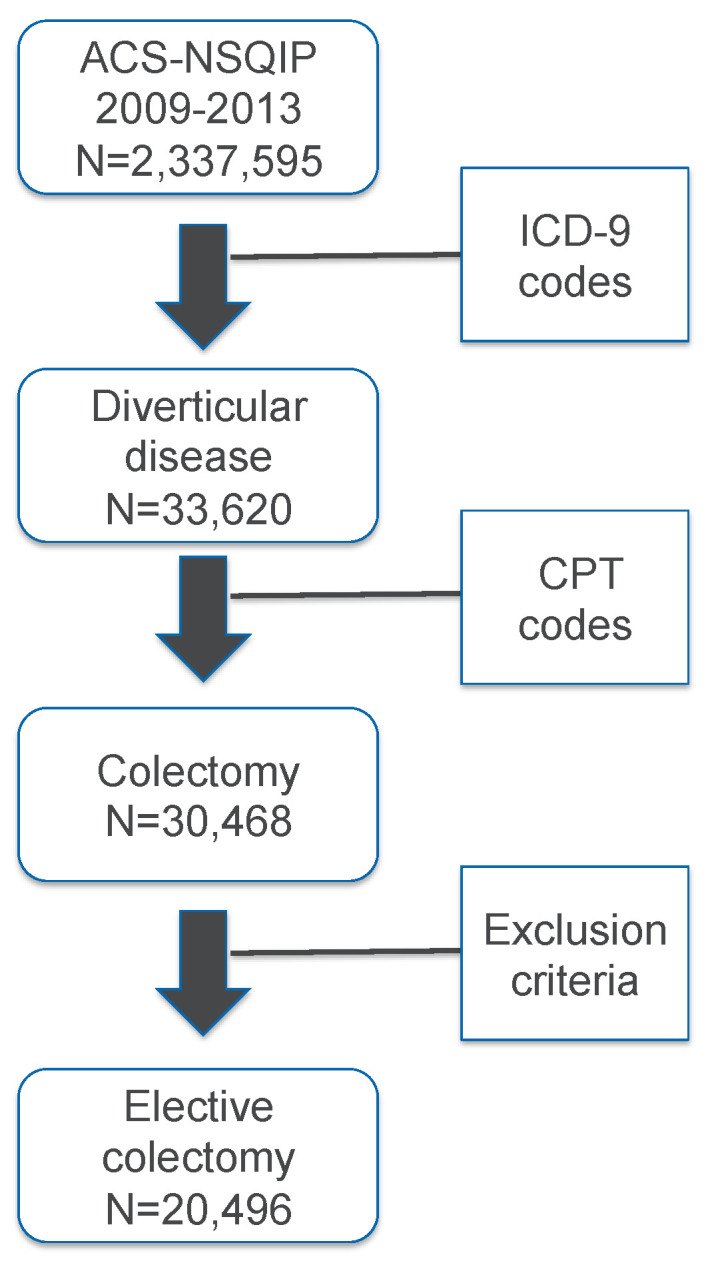
Patient selection process. ACS-NSQIP: American College of Surgeons National Surgical Quality Improvement Program; ICD-9: International Classification of Disease, 9th Revision; CPT: Current Procedural Terminology.

The database of the American College of Surgeons National Surgical Quality Improvement Program (ACS-NSQIP) from 2009 to 2013 was used in this study. Patients with diverticular disease were selected according to the International Classification of Disease, 9th Revision (ICD-9) diagnosis codes (Supplementary Materials). Open and laparoscopic colectomies were identified with the Current Procedural Terminology (CPT) codes (Supplementary Materials), and recorded as principal operative procedure, other procedure, or concurrent procedure. Elective colectomy patients were selected according to exclusion criteria, which included emergent operation, American Society of Anesthesiologists (ASA) class IV and V, dirty wound class, dyspnea at rest, total dependence of functional healthy status, colorectal cancer, preoperative systemic sepsis, and ventilator dependence. Figure 1 shows the flow diagram of the patient selection process.

### 2.2. Postoperative Outcomes

Postoperative outcomes included postoperative morbidities, 30-day mortality, and overall complication. The analyzed postoperative morbidities were surgical site infection (superficial, deep and organ), urinary tract infection, wound disruption, pneumonia, re-intubation, ventilator use for more than 48 h, pulmonary embolism, deep vein thrombosis, progressive renal insufficiency, acuter renal failure, stroke, cardiopulmonary resuscitation, myocardial infarction, blood transfusion, sepsis, septic shock, and return to operating room. The Accordion Severity Grading System was used to grade and weight the complications, mortality, and morbidities [21,22]. An overall complication score was calculated for each patient as the sum of the weighted scores of the complications that occurred in the patient.

### 2.3. Statistical Analyses

The Chi-squared test was used for univariate associations between groups and categorical variables. Univariate regression analyses were computed for continuous dependent variables, including overall complication and length of total hospital stay. Multiple regression analyses were used to demonstrate the associations between postoperative outcomes and adjusted for significant preoperative risk factors. A *p* value of <0.05 was considered significant. All analyses were performed using SPSS version 22, IBM, Armonk, NY USA.

## 3. Results

Of the 30,468 patients who were diagnosed with diverticular disease, 20,496 (67%) had an elective and 9972 (33%) had a non-elective colectomy. Table 1 shows the significant differences in their clinical demographics including age, race, gender, BMI, and other comorbidities. Patients who underwent elective colectomies were younger and had less medical comorbidity. The percentage of white patients in the elective colectomy group (86.2%) was higher than those in the non-elective colectomy group (82.4%), and the opposite was significant in patients of other races. Obesity (BMI ≥ 30) was more prevalent in the elective colectomy group.

The rate of 30-day mortality was 0.2% in the elective colectomy group (Table 2). Following the colectomy for diverticulitis, 18.7% of the elective colectomy group experienced at least one post-operative morbidity. Superficial surgical site infection was the most common postoperative morbidity (7.2%).

Univariate analyses of the risk factors associated with overall complications in elective colectomy are displayed in Table 3. Gender, ascites, and acute renal failure were not significantly associated with overall complication. After adjustment of the multiple regression analyses, the risk factors associated with overall complications included age ≥ 75, obesity, smoking, dyspnea, hypertension, dialysis, chronic steroid use, ASA III, and open colectomy (Table 3).

The average rate of the use of the laparoscopic approach in elective colectomy was 67.5% and this increased annually. There were significantly fewer overall complications, and a shorter length of total hospital stays in the laparoscopic group compared with the open procedure group (*p* < 0.001) (Table 4). Multiple regression analyses of the risk factors in laparoscopic colectomy patients demonstrated that age ≥ 75, smoking, hypertension, chronic steroid use, and ASA III were still significant risk factors for overall complications (Table 5).

## 4. Discussion

Using the large, nationally validated, and multi-institutional database of the ACS-NSQIP, we analyzed the postoperative outcomes and the associated risk factors in patients with diverticular disease receiving an elective colectomy, including via laparoscopy. In addition to the number of diverticulitis episodes, the pre-operative assessment of individual risk factors may be used as additional criteria to determine which patients with diverticular disease would not be suitable for an elective colectomy.

Although obesity (BMI ≥ 30) was previously not considered a significant risk factor for postoperative complications in general elective surgery [23] and major intra-abdominal cancer surgery [24], other studies have shown that obesity is an independent risk factor for postoperative complications in laparoscopic colectomies [25] and proctectomies [26]. In our study, obesity was significantly associated with overall complications during elective colectomy. Weight loss before an elective colectomy for diverticular disease is recommended to prevent postoperative complications in this high-risk population.

Preoperative smoking status is associated with postoperative complications in many kinds of surgical procedures [27,28]. Smoking is the leading preventable cause of morbidities [29]. Randomized trials have demonstrated that preoperative smoking cessation effectively reduces the postoperative complications following an elective hip or knee alloplasty [30], primary hernia repair, and laparoscopic cholecystectomies [31]. Systematic reviews concluded that postoperative morbidity was lower after intensive smoking cessation [32]. For colorectal surgery, previous trials showed that smoking cessation did not affect postoperative complications [33]. However, the study design featured a shorter period of intervention (2–3 weeks) and most of the patients included in this study had malignant neoplasm. Our study showed that smoking is an independent risk factor associated with postoperative complication in elective colectomy and in the laparoscopic subgroup for diverticular disease. More smoking cessation programs would decrease healthcare costs and postoperative complication rates [34].

Due to the high risk of perforation in recurrent diverticulitis, the immunocompromised patients undergoing steroid treatment or patients with chronic renal failure may benefit from an early elective resection after conservative treatments for diverticulitis [12,35]. However, a study of immunosuppressed patients with diverticulitis showed that they had higher mortality rates than non-immunosuppressed patients only during their first episode with a higher need for emergent operation. After the success of medical therapy for acute diverticulitis, the immunosuppressed patients were not good candidates for elective colectomy because the risk of recurrence and possibilities of further emergent surgery are similar to that of the patients without immunosuppression [35]. Compared with immunocompetent patients, immunosuppressed patients were at higher risk for major morbidities and wound dehiscence [36]. In our analyses, the patients undergoing chronic steroid treatment or dialysis also had more postoperative complications after elective colectomy. Elective colectomy in immunocompromised patients with diverticular disease should be given careful consideration [37].

A laparoscopic surgical approach for elective colectomy improved postoperative outcomes [38] and has been associated with lower 30-day morbidity compared with an open colectomy [39]. But there are still many risk factors associated with complications after laparoscopic procedures [40]. A study of 526 patients who underwent laparoscopic colectomy for recurrent diverticulitis showed postoperative complications were associated with some risk factors including age ≥ 75, previous myocardial infarction and heart failure [41]. We have also demonstrated more independent preoperative risk factors, which should be recognized and considered by the surgeon to avoid as many complications as possible.

Compared with white patients, in our study, patients of other races had higher rates of non-elective colectomy for diverticular disease. Schneider et al. reported a similar conclusion of differential association of race with treatment in Medicare patients undergoing diverticulitis surgery [42]. Black patients received urgent or emergency surgery more often than white patients did. Lower socioeconomic status and lack of health insurance were thought to be powerful predictors of diverticulitis severity and treatment [43]. Differences in patient preference and physician’s recommendations may also be partly responsible [44]. Further studies should be designed to elucidate the independent factors involved in this racial disparity.

In our study, the score for overall complications was calculated according to the numbers and severity grade of postoperative mortality and 19 morbidities occurred in individual patients. We transferred the overall complications, mortality, and morbidities, which are discrete variables, into a continuous variable which reflects the total effect of complications that occurred in every patient. It was more complicated and accurate to identify the independent risk factors of overall complication.

Diabetes mellitus has been reported as significant risk factor in surgical site infection [45] and anastomosis leakage [46] in colorectal surgery. In a meta-analysis, diabetes mellitus was associated with postoperative urinary tract injection and hospital readmissions following colorectal surgery. However, the other complications, including intra-abdominal infections, wound dehiscence, pulmonary complications, reoperation, etc., did not increase in patients with diabetes mellitus [47]. In our study, overall complications included postoperative mortality and 19 morbidities, which are not all associated with diabetes mellitus. This may be the reason why diabetes mellitus was not identified as a risk factor for postoperative overall complications in our multivariant analysis,

Like other studies based on the ACS-NSQIP database, the limitations of this study are those inherent to utilizing a large data set across a broad range of surgical practices, and some insufficiency in operation-specific analyses. The database does not include clinical information on the presenting disease, such as previous episodes of acute diverticulitis, the severity of diverticulitis, or the indications for colectomy [36]. In addition to emergency operation, we adopted other associated exclusion criteria to reduce the selection bias. Additionally, the ACS-NSQIP database records only those complications that occur in the 30-day postoperative period and therefore outcomes after 30 days are not available. Finally, specific events related to colectomy, such as type of anastomosis, leakage, postoperative ileus, and the detail of diverting stoma were not listed in the ACS-NSQIP database and were not included in the postoperative complications considered in our study.

## 5. Conclusions

Before suggesting an elective colectomy for patients recovering from acute episodes of diverticulitis, using individualized evaluation and correcting any associated preoperative risk factors demonstrated in our study would improve individual outcomes and reduce postoperative complications. Clinical trials should be designed to compare the differences in postoperative complications between the groups with or without reducing the risk factors identified in this study before performing an elective colectomy for diverticular disease.

## Figures and Tables

**Table 1:** Clinical demographics of patients who underwent colectomy.

Characteristics	Elective Colectomy (*n* = 20,496)	Non-Elective Colectomy (*n* = 9972)	*p* Value
Age			
<65	14,355 (70.0)	5466 (54.8)	<0.001
65–74	4266 (20.8)	2292 (23.0)	
75–84	1632 (8.0)	1648 (16.5)	
>84	243 (1.2)	567 (5.7)	
Race			
White	17,667 (86.2)	8221 (82.4)	<0.001
Black	1241 (6.1)	850 (8.5)	
Asian	189 (0.9)	137 (1.4)	
Native Hawaiian or Pacific Islander	39 (0.2)	35 (0.4)	
Other/missing	1263 (6.2)	678 (6.8)	
Gender (female)	11,279 (55.0)	5202 (52.2)	<0.001
BMI ≥ 30 (kg/m^2^)	8200 (40.0)	3864 (38.7)	0.034
DM			
No	27,213 (89.3)	8594 (86.2)	<0.001
Oral	2414 (7.9)	912 (9.1)	
Insulin	842 (2.8)	467 (4.7)	
Smoking	4238 (20.7)	2350 (23.6)	<0.001
COPD	601 (2.9)	860 (8.6)	<0.001
Ascites	16 (0.1)	130 (1.3)	<0.001
Chronic heart failure	26 (0.1)	199 (2.0)	<0.001
Hypertension	9143 (44.6)	5463 (54.8)	<0.001
Acute renal failure	12 (0.1)	99 (1.0)	<0.001
Dialysis	66 (0.3)	173 (1.7)	<0.001
Chronic steroid use	530 (2.6)	1019 (10.2)	<0.001

DM, diabetes mellitus; BMI, body mass index; COPD, chronic obstructive pulmonary disease. Values in parentheses are percentages.

**Table 2:** The ratio of postoperative mortality and morbidities in elective colectomy patients (*n* = 20,496).

Postoperative Outcomes	Number	Ratio (%)
30-day mortality	42	0.2
Superficial surgical site infection	1481	7.2
Return to operating room	847	4.1
Transfusion	684	3.3
Organ surgical site infection	642	3.1
Sepsis	510	2.5
Urinary tract infection	432	2.1
Deep surgical site infection	225	1.1
Wound disruption	218	1.1
Pulmonary embolism	191	0.6
Deep vein thrombus	114	0.6
Pneumonia	130	0.6
Re-intubation	105	0.5
Ventilator > 48 h	104	0.5
Septic shock	102	0.5
Progressive renal insufficiency	90	0.4
Acute renal failure	46	0.2
Myocardial infection	49	0.2
Stroke	20	0.1
Cardiopulmonary resuscitation	26	0.1

**Table 3:** Univariate and multiple regression analyses for overall complications in elective colectomy patients.

Risk Factors	Univariate B (95% CI)	Multiple B (95% CI)
Age <65	Reference	Reference
65–74	0.017 (0.007–0.028) *	0.005 (−0.006–0.016)
75–84	0.077 (0.062–0.093) **	0.052 (0.036–0.069) **
>84	0.11 (0.071–0.149) **	0.076 (0.037–0.116) **
Gender, (female)	0.005 (−0.003–0.014)	-
BMI ≥ 30 (kg/m^2^)	0.024 (0.015–0.032) **	0.022 (0.013–0.031) **
DM No	Reference	-
Oral	0.022 (0.006–0.038) *	-
Insulin	0.081 (0.05–0.113) **	-
Smoking	0.022 (0.012–0.033) **	0.023 (0.013–0.034) **
COPD	0.074 (0.049–0.099) **	-
Dyspnea	0.081 (0.061–0.101) **	0.044 (0.024–0.064) **
Ascites	0.06 (–0.092–0.211)	-
Chronic heart failure	0.144 (0.025–0.262) *	-
Hypertension	0.04 (0.032–0.049) **	1.013 (0.863–1.163) **
Acute renal failure	−0.029 (–0.2–0.145)	0.014 (0.005–0.024) *
Dialysis	0.205 (0.13–0.279) **	0.141 (0.067–0.215) **
Chronic steroid use	0.118 (0.092–0.145) **	0.085 (0.058–0.111) **
FS Independent	Reference	-
Partially dependent	0.088 (0.04–0.137) **	-
ASA I	Reference	Reference
II	0.021 (0–0.042)	0.002 (−0.019–0.024)
III	0.087 (0.065–0.109) **	0.035 (0.012–0.058) *
Wound II	Reference	-
III	0.019 (0.008–0.029) **	-
OP Laparoscopic	Reference	Reference
Open	0.071 (0.062–0.08) **	0.055 (0.046–0.64) **

BMI, body mass index; DM, diabetes mellitus; COPD, chronic obstructive pulmonary disease; FS, functional status; ASA, American Society of Anesthesiologists; Wound II, clean/contaminated; Wound III, contaminated; OP, operation. *: *p* value < 0.05; **: *p* value < 0.001.

**Table 4:** Simple regression analysis for overall complications and length of total hospital stays between laparoscopic and open approach.

Variables	Mean	B (Coefficient)	95% CI	*p* Value
Overall complication				
Laparoscopic	0.071	0		
Open	0.142	0.071	0.062–0.08	<0.001
Length of total hospital stay				
Laparoscopic	4.794	0		
Open	7.786	2.992	2.827–3.157	<0.001

**Table 5:** Multiple regression analysis for overall complications in laparoscopic colectomy patients.

Risk Factors	Overall Complication B (95% CI)
Age <65	Reference
65–74	0.05 (−0.007–0.017)
75–84	0.028 (0.008–0.047) *
>84	0.109 (0.059–0.16) **
Smoking	0.032 (0.021–0.044) **
Dyspnea	0.046 (0.023–0.069) **
Hypertension	0.013 (0.003–0.023) *
Chronic steroid use	0.057 (0.026–0.088) **
ASA I	Reference
II	0.006 (−0.015–0.027)
III	0.031 (0.008–0.054) *

ASA, American Society of Anesthesiologists. *: *p* value < 0.05; **: *p* value < 0.001.

## Data Availability

Data is contained within the article or Supplementary Materials.

## Supplementary Materials

The following supporting information can be downloaded at: https://www.mdpi.com/article/10.3390/jcm12237338/s1, Table S1: Revision (ICD-9) diagnosis codes and Current Procedural Terminology (CPT) codes.

## References

[1] Peery A.F., Dellon E.S., Lund J., Crockett S.D., McGowan C.E., Bulsiewicz W.J., Gangarosa L.M., Thiny M.T., Stizenberg K., Morgan D.R. (2012). Burden of gastrointestinal disease in the United States: 2012 update. Gastroenterology.

[2] Etzioni D.A., Mack T.M., Beart R.W., Kaiser A.M. (2009). Diverticulitis in the United States: 1998–2005: Changing patterns of disease and treatment. Ann. Surg..

[3] Sandler R.S., Everhart J.E., Donowitz M., Adams E., Cronin K., Goodman C., Gemmen E., Shah S., Avdic A., Rubin R. (2002). The burden of selected digestive diseases in the United States. Gastroenterology.

[4] Stollman N., Raskin J.B. (2004). Diverticular disease of the colon. Lancet.

[5] Jacobs D.O. (2007). Clinical practice. Diverticulitis. N. Engl. J. Med..

[6] Salem L., Veenstra D.L., Sullivan S.D., Flum D.R. (2004). The timing of elective colectomy in diverticulitis: A decision analysis. J. Am. Coll. Surg..

[7] Rose J., Parina R.P., Faiz O., Chang D.C., Talamini M.A. (2015). Long-term Outcomes After Initial Presentation of Diverticulitis. Ann. Surg..

[8] Roberts P., Abel M., Rosen L., Cirocco W., Fleshman J., Leff E., Levien D., Pritchard T., Wexner S., Hicks T. (1995). Practice parameters for sigmoid diverticulitis. The Standards Task Force American Society of Colon and Rectal Surgeons. Dis. Colon Rectum.

[9] Wong W.D., Wexner S.D., Lowry A., Vernava A., Burnstein M., Denstman F., Fazio V., Kerner B., Moore R., Oliver G. (2000). Practice parameters for the treatment of sigmoid diverticulitis—Supporting documentation. The Standards Task Force. The American Society of Colon and Rectal Surgeons. Dis. Colon Rectum.

[10] Masoomi H., Buchberg B.S., Magno C., Mills S.D., Stamos M.J. (2011). Trends in diverticulitis management in the United States from 2002 to 2007. Arch. Surg..

[11] Janes S., Meagher A., Frizelle F.A. (2005). Elective surgery after acute diverticulitis. Br. J. Surg..

[12] Chapman J., Davies M., Wolff B., Dozois E., Tessier D., Harrington J., Larson D. (2005). Complicated diverticulitis: Is it time to rethink the rules?. Ann. Surg..

[13] Thaler K., Baig M.K., Berho M., Weiss E.G., Nogueras J.J., Arnaud J.P., Wexner S.D., Bergamaschi R. (2003). Determinants of recurrence after sigmoid resection for uncomplicated diverticulitis. Dis. Colon Rectum.

[14] Van Arendonk K.J., Tymitz K.M., Gearhart S.L., Stem M., Lidor A.O. (2013). Outcomes and costs of elective surgery for diverticular disease: A comparison with other diseases requiring colectomy. JAMA Surg..

[15] Rafferty J., Shellito P., Hyman N.H., Buie W.D. (2006). Practice parameters for sigmoid diverticulitis. Dis. Colon Rectum.

[16] Feingold D., Steele S.R., Lee S., Kaiser A., Boushey R., Buie W.D., Rafferty J.F. (2014). Practice parameters for the treatment of sigmoid diverticulitis. Dis. Colon Rectum.

[17] Kopylov U., Ben-Horin S., Lahat A., Segev S., Avidan B., Carter D. (2012). Obesity, metabolic syndrome and the risk of development of colonic diverticulosis. Digestion.

[18] Milovanovic T., Pantic I., Dragasevic S., Lugonja S., Dumic I., Rajilic-Stojanovic M. (2021). The Interrelationship among Non-Alcoholic Fatty Liver Disease, Colonic Diverticulosis and Metabolic Syndrome. J. Gastrointest. Liver Dis..

[19] Pantic I., Lugonja S., Rajovic N., Dumic I., Milovanovic T. (2021). Colonic Diverticulosis and Non-Alcoholic Fatty Liver Disease: Is There a Connection?. Medicina.

[20] Piessen G., Muscari F., Rivkine E., Sbai-Idrissi M.S., Lorimier G., Fingerhut A., Dziri C., Hay J.M. (2011). Prevalence of and risk factors for morbidity after elective left colectomy: Cancer vs. noncomplicated diverticular disease. Arch. Surg..

[21] Strasberg S.M., Linehan D.C., Hawkins W.G. (2009). The accordion severity grading system of surgical complications. Ann. Surg..

[22] Vollmer C.M., Lewis R.S., Hall B.L., Allendorf J.D., Beane J.D., Behrman S.W., Callery M.P., Christein J.D., Drebin J.A., Epelboym I. (2015). Establishing a quantitative benchmark for morbidity in pancreatoduodenectomy using ACS-NSQIP, the Accordion Severity Grading System, and the Postoperative Morbidity Index. Ann. Surg..

[23] Dindo D., Muller M.K., Weber M., Clavien P.A. (2003). Obesity in general elective surgery. Lancet.

[24] Mullen J.T., Davenport D.L., Hutter M.M., Hosokawa P.W., Henderson W.G., Khuri S.F., Moorman D.W. (2008). Impact of body mass index on perioperative outcomes in patients undergoing major intra-abdominal cancer surgery. Ann. Surg. Oncol..

[25] Mustain W.C., Davenport D.L., Hourigan J.S., Vargas H.D. (2012). Obesity and laparoscopic colectomy: Outcomes from the ACS-NSQIP database. Dis. Colon Rectum.

[26] Smith R.K., Broach R.B., Hedrick T.L., Mahmoud N.N., Paulson E.C. (2014). Impact of BMI on postoperative outcomes in patients undergoing proctectomy for rectal cancer: A national surgical quality improvement program analysis. Dis. Colon Rectum.

[27] Sharma A., Deeb A.P., Iannuzzi J.C., Rickles A.S., Monson J.R., Fleming F.J. (2013). Tobacco smoking and postoperative outcomes after colorectal surgery. Ann. Surg..

[28] Gronkjaer M., Eliasen M., Skov-Ettrup L.S., Tolstrup J.S., Christiansen A.H., Mikkelsen S.S., Becker U., Flensborg-Madsen T. (2014). Preoperative smoking status and postoperative complications: A systematic review and meta-analysis. Ann. Surg..

[29] Khullar D., Maa J. (2012). The impact of smoking on surgical outcomes. J. Am. Coll. Surg..

[30] Moller A.M., Villebro N., Pedersen T., Tonnesen H. (2002). Effect of preoperative smoking intervention on postoperative complications: A randomised clinical trial. Lancet.

[31] Lindstrom D., Sadr Azodi O., Wladis A., Tonnesen H., Linder S., Nasell H., Ponzer S., Adami J. (2008). Effects of a perioperative smoking cessation intervention on postoperative complications: A randomized trial. Ann. Surg..

[32] Thomsen T., Tonnesen H., Moller A.M. (2009). Effect of preoperative smoking cessation interventions on postoperative complications and smoking cessation. Br. J. Surg..

[33] Sorensen L.T., Jorgensen T. (2003). Short-term pre-operative smoking cessation intervention does not affect postoperative complications in colorectal surgery: A randomized clinical trial. Color. Dis..

[34] Gaskill C.E., Kling C.E., Varghese T.K., Veenstra D.L., Thirlby R.C., Flum D.R., Alfonso-Cristancho R. (2017). Financial benefit of a smoking cessation program prior to elective colorectal surgery. J. Surg. Res..

[35] Biondo S., Borao J.L., Kreisler E., Golda T., Millan M., Frago R., Fraccalvieri D., Guardiola J., Jaurrieta E. (2012). Recurrence and virulence of colonic diverticulitis in immunocompromised patients. Am. J. Surg..

[36] Al-Khamis A., Abou Khalil J., Demian M., Morin N., Vasilevsky C.A., Gordon P.H., Boutros M. (2016). Sigmoid Colectomy for Acute Diverticulitis in Immunosuppressed vs Immunocompetent Patients: Outcomes from the ACS-NSQIP Database. Dis. Colon Rectum.

[37] Binda G.A., Cuomo R., Laghi A., Nascimbeni R., Serventi A., Bellini D., Gervaz P., Annibale B. (2015). Practice parameters for the treatment of colonic diverticular disease: Italian Society of Colon and Rectal Surgery (SICCR) guidelines. Tech. Coloproctol..

[38] Russ A.J., Obma K.L., Rajamanickam V., Wan Y., Heise C.P., Foley E.F., Harms B., Kennedy G.D. (2010). Laparoscopy improves short-term outcomes after surgery for diverticular disease. Gastroenterology.

[39] Kakarla V.R., Nurkin S.J., Sharma S., Ruiz D.E., Tiszenkel H. (2012). Elective laparoscopic versus open colectomy for diverticulosis: An analysis of ACS-NSQIP database. Surg. Endosc..

[40] Kirchhoff P., Dincler S., Buchmann P. (2008). A multivariate analysis of potential risk factors for intra- and postoperative complications in 1316 elective laparoscopic colorectal procedures. Ann. Surg..

[41] Kirchhoff P., Matz D., Dincler S., Buchmann P. (2011). Predictive risk factors for intra- and postoperative complications in 526 laparoscopic sigmoid resections due to recurrent diverticulitis: A multivariate analysis. World J. Surg..

[42] Schneider E.B., Haider A., Sheer A.J., Hambridge H.L., Chang D.C., Segal J.B., Wu A.W., Lidor A.O. (2011). Differential association of race with treatment and outcomes in Medicare patients undergoing diverticulitis surgery. Arch. Surg..

[43] Lidor A.O., Gearhart S.L., Wu A.W., Chang D.C. (2008). Effect of race and insurance status on presentation, treatment, and mortality in patients undergoing surgery for diverticulitis. Arch. Surg..

[44] Lucas F.L., Stukel T.A., Morris A.M., Siewers A.E., Birkmeyer J.D. (2006). Race and surgical mortality in the United States. Ann. Surg..

[45] Amri R., Dinaux A.M., Kunitake H., Bordeianou L.G., Berger D.L. (2017). Risk Stratification for Surgical Site Infections in Colon Cancer. JAMA Surg..

[46] Penna M., Hompes R., Arnold S., Wynn G., Austin R., Warusavitarne J., Moran B., Hanna G.B., Mortensen N.J., Tekkis P.P. (2019). Incidence and Risk Factors for Anastomotic Failure in 1594 Patients Treated by Transanal Total Mesorectal Excision: Results from the International TaTME Registry. Ann. Surg..

[47] Tan D.J.H., Yaow C.Y.L., Mok H.T., Ng C.H., Tai C.H., Tham H.Y., Foo F.J., Chong C.S. (2021). The influence of diabetes on postoperative complications following colorectal surgery. Tech. Coloproctol..

