# Supplementary material for: Risk Factors Associated with Postoperative Outcomes in Diverticular Disease Undergoing Elective Colectomy—A Retrospective Cohort Study from the ACS-NSQIP Database
Source: J Clin Med. 2023 Nov 27;12(23):7338. doi: 10.3390/jcm12237338 (PMC10707174; doi:10.3390/jcm12237338)
Supplement: Supplementary file 1 [file jcm-12-07338-s001.zip › jcm-2676964-supplementary.pdf]

(A) International Classification of Disease, 9th Revision (ICD-9) diagnosis codes:

562.1: Diverticulosis of colon without hemorrhage

562.11: Diverticulitis of colon without hemorrhage

562.12: Diverticulosis of colon with hemorrhage

562.13: Diverticulitis of colon with hemorrhage

(B) Current Procedural Terminology (CPT) codes:

Open colectomy: 44140, 44141, 44143, 44144, 44145, 44146, 44147, 44150, 44151, 44155,44156, 44157, 44158, 44160

Laparoscopic colectomy: 44204, 44205, 44206, 44207, 44208, 44210, 44211, 44212
